# Cleft Care UK study. Part 5: child psychosocial outcomes and satisfaction with cleft services

**DOI:** 10.1111/ocr.12113

**Published:** 2015-11-16

**Authors:** A Waylen, A R Ness, A K Wills, M Persson, N Rumsey, J R Sandy

**Affiliations:** School of Oral and Dental Sciences, University of BristolBristol, UK; National Institute for Health Research (NIHR) Biomedical Research Unit in Nutrition, Diet and Lifestyle at the University Hospitals Bristol NHS Foundation Trust and the University of BristolBristol, UK; Centre for Appearance Research, University of the West of EnglandBristol, UK

**Keywords:** cleft lip, cleft palate, personal satisfaction, treatment outcome

## Abstract

**Objectives:**

To describe the impact of cleft service centralization on parental perceptions of child outcomes and satisfaction with care from the Cleft Care UK (CCUK) study and compare them to the Clinical Standards Advisory Group (CSAG) study that took place 15 years earlier.

**Setting and Sample Population:**

A subgroup of respondents from a UK multicentre cross-sectional study (CCUK) of 5-year-olds born with non-syndromic unilateral cleft lip and palate.

**Materials and Methods:**

Data on parents’ perceptions of child self-confidence and their satisfaction with treatment outcomes and service provision were collected via self-report questionnaires. Data were compared with findings from the 1998 CSAG study.

**Results:**

Fewer parents in the CCUK study perceived their children as having poor self-confidence than in the 1998 CSAG study (8 and 19%, respectively). At least 81% of parents report satisfaction with the child’s facial features after surgery and 98% report being satisfied with the care received. These results are similar to those reported in 1998. There is no evidence of an adverse impact on families’ ability to attend appointments at the cleft clinic following centralization. Levels of reported problems (around 30%) with attendance were similar to those reported by CSAG.

**Conclusion:**

Centralization of cleft services appears to have improved parental perceptions of some child outcomes but has made little difference to already high levels of parental satisfaction with cleft care services. Centralization is not associated with an increase in the proportion of families who find it difficult to attend appointments.

## Introduction

Every year, in the United Kingdom (UK), around 1:700 infants are born with a cleft lip and/or palate [CLP] 1,2. This condition is associated with adverse physical outcomes for the child such as poor facial growth and dental anomalies 3,4 and communication issues such as speech disorders and poor hearing 5–7. It is well known that, from childhood onwards, attributions about an individual’s character, personality, academic performance and social relationships are based, at least in part, on his or her appearance and perceived attractiveness 8,9. For children born with CLP, there is evidence that impaired facial growth and dental anomalies are associated with adverse psychosocial outcomes 1,10 including low self-confidence 11, an increased risk of being teased and bullied 12 and problematic social relationships 13,14. Treatment for CLP is undertaken with the aim of achieving the best aesthetic result and optimizing function in terms of hearing, feeding and speech 1, but it does require multiple surgical and dental interventions across infancy, childhood and adulthood.

In 1998, a study commissioned by the Clinical Standards Advisory Group (CSAG) examined the process of care, treatment and outcomes for children born with CLP. This research comprised surveys of parents of 5- and 12-year-old children who had received treatment for a unilateral cleft lip and palate (UCLP). Parental perceptions of key psychosocial outcomes were assessed using a self-report questionnaire developed by the Royal College of Surgeons of England Steering Group [RCS] 8. Questionnaire items asked about parental perceptions of the child’s self-confidence, their satisfaction with the child’s facial appearance (teeth, lips, nose, profile, hearing and speech) and also with the level of care provided by the cleft service. Nineteen per cent of parents believed that the cleft had a negative impact on their 5-year-old child’s self-confidence, but the vast majority (93%) also reported that the care and attention they had received within the cleft service and associated treatment and outcomes were either good or excellent 1.

The 1998 CSAG report also presented findings from a survey undertaken by the Cleft Lip and Palate Association (CLAPA) in 1996 1. Data were collected from 102 parents of children aged 4 years and under about their satisfaction with the service and treatment provided. Most parents were satisfied with the service they and their child received, but there were concerns around a lack of communication with service providers and also provision of information about cleft lip and palate and its associated treatment.

Having taken into account the findings from the cross-sectional survey of children with cleft lip and palate and the CLAPA survey of parental opinion, the key recommendation from the 1998 CSAG study was one of service centralization. Government was advised to reduce the 57 cleft services in the UK to between 8 and 15 centres in order to ensure high-quality clinical experience and a full range of readily available clinical services, including psychological support. Since 1998, this process of centralization has been ongoing and by 2011, eleven centres or managed clinical networks were providing cleft care for all children born with a cleft lip/palate in the UK 9.

There were two main aims to this Cleft Care UK (CCUK) study. The first was to examine the impact of centralization on parental reports of child self-confidence at 5 years of age and parental satisfaction with 1) the child’s facial appearance and 2) the provision of cleft services. This was performed by comparing these psychosocial outcomes from the 1998 study with findings from the current study. The second aim was to report parental perceptions on a broader range of measures not previously assessed in the 1998 CSAG study of 5-year-old children. These included parental perceptions of whether their child was experiencing teasing and/or bullying, whether parents felt they or their child would benefit from counselling services (someone to talk to about the cleft), additional difficulties in parents’ lives associated with the cleft and parental perceptions of their relationship with the cleft team.

## Subjects and methods

A cross-sectional questionnaire study was designed to replicate the investigation of child psychology and parental satisfaction undertaken in the 1998 CSAG study. This is described in detail in the methods paper in this series.

Briefly, 268 5-year-old children (67.5% male), born with non-syndromic UCLP between 1 April 2005 and 31 March 2007, were recruited from cleft centres across the UK. Ethical approval was obtained (REC reference number: 10/H0107/33, South West 5 REC) and included consent for extended psychosocial, health and lifestyle and economic questions. Eligible families were invited to attend a designated study clinic. Consent from parents to take part in the study and assent from the children themselves were sought on arrival at the clinic. Two questionnaires were used, one was concerned with psychosocial assessment of the child and the other with the health and lifestyle of the family.

The psychosocial assessment questionnaire was administered by a psychologist. The questionnaire used was a modified version of that used in the CSAG survey. These modifications reduced the number of items from 18 to 8. Parents were asked to complete the health and lifestyle questionnaires and items about satisfaction with the cleft service either while they were at the clinic or when they returned home.

### Questionnaire measures for comparison with the 1998 CSAG findings

The RCS questionnaire included items about whether parents felt that their child’s self-confidence had been affected by the cleft. Parents were also asked to rate how satisfied they were with the child’s appearance: specifically the teeth, lip, nose and profile and also their hearing and speech. Items about parental satisfaction with cleft service delivery and also provision of information about the child’s treatment were assessed using questions in a health and lifestyle questionnaire. This questionnaire included items about the time taken to travel to the clinic, problems associated with clinic attendance and parental satisfaction with services provided by the cleft team including the provision of information about treatment.

### Comparison of child’s self-confidence

Data on psychosocial and well-being concepts collected in both the CSAG and the CCUK studies are reported here. However, it is important to note that, in order to achieve consistency with the Cleft Psychologists’ newly developed 5-year-old audit protocols, items were not always presented in the same way in each questionnaire and response options sometimes differed. In the CSAG study, for the item asking whether the child’s self-confidence had been affected by the cleft, parents were asked to respond either yes or no; in the CCUK study, they were asked to rate their response on a scale from 0 to 10 where 0 = a very negative impact of the cleft on the child’s self-confidence, 5 = no difference and 10 = a very positive impact of the cleft. For the purposes of this study, parents were considered to have reported a negative impact on the child’s self-confidence if they responded with a score between 0 and 3.

### Comparison of parental satisfaction of appearance

In the CSAG study, parents were asked to rate whether they were dissatisfied, satisfied or very satisfied with their child’s appearance; in the CCUK study, parents were asked to rate their satisfaction with the child’s appearance on a 0–10 scale where 0 = very unhappy and 10 = very happy. As there was no anchor point for a score of five in the CCUK questionnaire, we classified those parents who rated their satisfaction as five or above as happy or very happy with the child’s appearance and those with scores of four or less as unhappy with the child’s appearance. To compare these data with the CSAG data, we have also aggregated the CSAG categories satisfied or very satisfied into a single category (see Table 2).

### Comparison of parental satisfaction with service provision

The CSAG study asked whether parental satisfaction with 1) the care and attention received and 2) treatment and outcome was poor, reasonable, good or excellent. The CCUK study asked parents to rate their satisfaction with the cleft service on a scale from 0 to 10: we have categorized scores of 0–2 as representing poor satisfaction, 3–5 as reasonable, 6–8 as good and 9–10 as excellent.

### Questionnaire measures from the health and lifestyles questionnaire not reported previously in CSAG

Parents were asked whether or not they felt that their child was teased or bullied because of their cleft (yes/no), and, if so, what was their child’s response to the teasing/bullying (yes/no to specific behaviours). They were also asked to what extent they believed the child was bothered by the teasing/bullying (reported on an 11-point Likert scale where 0 = bothered a great deal and 10 = not bothered at all) and whether the cleft made any difference to their own current well-being (11-point Likert scale: 0 = cleft makes me very unhappy, 5 = cleft makes no difference and 10 = cleft makes me very happy). Parents were asked whether it would be helpful for either the child or they themselves to have somebody to talk to about issues concerned with the cleft (yes, no or maybe at a later date). Another set of questions asked about the relationship between the family and the cleft team with responses given on 11-point Likert scales: how the child felt about coming to the clinic (0 = doesn’t like coming, 10 = likes coming) and how nervous the parents felt about seeing the cleft team (0 = very nervous, 10 = not nervous). Finally, they were asked about the extent to which they believed they were involved in the decision-making process about the child’s treatment (0 = not at all, 10 = very involved).

### Statistical analysis

Proportions, medians and interquartile range statistics were calculated in the CCUK sample and, where available, are also reported for the CSAG group.

## Results

All parents attending the clinic appointment with their child were asked to complete the psychosocial assessment questionnaire, and completed questionnaires were returned by 246 parents (92% of the recruited sample). Data about satisfaction with service and travelling to and from appointments were collected within the health and lifestyles questionnaire that was given to parents with the option of completing it in clinic or at home. These questionnaires were completed and returned by 140 families (52% of the recruited sample).

### Comparison of current findings with those collected in the 1998 CSAG study

Data about parental perceptions of child self-confidence and also satisfaction with the child’s appearance, speech and hearing were collected during the clinic and responses to these items were provided by the majority of families who took part in the study. In 1998, 19% of parents reported that their 5-year-old child’s self-confidence had been adversely affected by their cleft, but this was true for only 8% of parents in the recent CCUK study (reporting a score of ≤3 for this item).

### Satisfaction with child’s appearance

In both, the 1998 CSAG study and the current CCUK study, at least 81% of parents reported that they were satisfied with the appearance of different elements of their child’s face (*p* < 0.001) (see Table1).

**Table 1:** Parent’s satisfaction with the child’s appearance, speech and hearing after surgery: CCUK and CSAG (data as reported by CSAG 1)

	CCUK		CSAG (5-year old children)
	% scoring ≥5 (0 = very unhappy, 10 = very happy)		% satisfied or very satisfied
	*N* (%)	Median (IQR)	*N* (%)
Appearance of teeth	212/246 (86)	7 (6,7)	185/229 (81)
Appearance of lip	234/246 (95)	9 (8,9)	217/229 (95)
Appearance of nose	214/247 (87)	8 (6,8)	209/229 (91)
Speech	226/246 (92)	8 (7,8)	217/229 (95)
Hearing	220/246 (89)	9 (7,10)	210/229 (92)
Profile	236/245 (96)	9 (8,9)	221/229 (97)

### Satisfaction with service

In the current survey, 98% of parents reported that the service they had received from the cleft team had been good or excellent, whereas 93% reported good or excellent care and attention in the CSAG study; 89% of those who participated in the CSAG study reported that their satisfaction with the treatment received was good or excellent, but this aspect of satisfaction was not assessed in the current study. With regard to items asked in the CLAPA survey that were summarized in the CSAG report, 75% of parents in the CCUK study reported that they had been given enough information about their child’s treatment. In the CLAPA survey, parents expressed concerns related to a lack of information for parents especially around feeding and pain control and a lack of communication within departments. Seventy-four per cent of CCUK respondents reported that explanations given by team members were very easy to understand; one in three CLAPA respondents were unhappy with the way they were told about their child’s cleft and they expressed concern about a lack of up-to-date information about clefts and their treatment. Seventy-five per cent of CCUK respondents reported that the cleft team members understood their concerns and 97% responded ‘yes’ when asked if they were getting enough support from the team. CLAPA respondents reported that some staff were insensitive and lacking in understanding regarding the impact of a diagnosis of cleft, and some were concerned about the lack of aftercare following the child’s operation and also the lack of continuity of care. (See Table2 for medians and IQR statistics for CCUK data).

**Table 2:** Parental reports of provision of information and satisfaction with service (CCUK study)

	Median (IQR)
Have you been given enough information about your son/daughter’s treatment? (*N* = 139)	10 (8,10)
(0 = never, 10 = always)	
How do you find the explanations given by members of the team? (*N* = 139)	9 (8,10)
(0 = very difficult to understand, 10 = very easy to understand)	
Do you feel that members of the team understand your concerns? (*N* = 139)	10 (9,10)
(0 = never, 10 = always)	
Overall, how satisfied are you with the service you have had from the cleft team? (*N* = 138)	10 (9,10)
(0 = not at all satisfied, 10 = very satisfied)	

Parents were also asked how long it took them to travel from home to the cleft clinic: in response to the current CCUK survey, 32% of parents said it took them longer than one hour to get to the clinic compared to 30% of parents who took part in the 1998 CSAG study. In the CCUK study, 31% of parents reported difficulties in attending for other reasons including taking time off work, arranging child care for siblings and the child missing school compared to 36% in the CSAG study.

### Findings from the current CCUK study not reported previously

When asked whether they felt their 5-year-old child had been exposed to teasing or bullying about the cleft, 24 parents (10%) said yes. Of these, 17 parents said that they felt that their child was bothered by this behaviour and 15 reported that the child got upset. Twenty-three of the parents said that their child always told someone if they had been bullied (see Table3).

**Table 3:** Child behaviour associated with teasing and bullying (psychosocial assessment questionnaire for CCUK study)

	Response	*N* (%)
Teasing or bullying is currently a problem for the child	Yes	24/248 (10%)
Child bothered by teasing/bullying	Score <5	17/23 (74%)
Child gets upset	Yes	15/24 (63%)
Child gets angry	Yes	10/24 (42%)
Child ignores it	Yes	8/24 (33%)
Child tells someone	Yes	23/24 (96%)
Child uses antibullying strategies	Yes	0/24

When asked if they or their child would find it helpful to talk to somebody about issues concerned with the cleft, 3% of parents confirmed that they themselves would find it helpful and 6% that their child would find it helpful. Thirty-six per cent of parents said they might want to talk to someone at a later date and 55% said that their child might find it helpful to be able to speak to somebody in the future.

When asked how their child felt about coming to the clinic, 24% of parents reported a score of 5 or less on an 11-point scale (0 = the child doesn’t like coming, 10 = the child likes coming. With respect to their own feelings, 21% scored ≤5 (0 = very nervous, 10 = not nervous). Only 4% of parents recorded a score lower than the median (suggesting a lack of involvement) when asked whether they felt they were involved in the decision-making process about their child’s treatment (see Table4 for medians and IQRs).

**Table 4:** Relationships between the family and the cleft team

	Median (IQR)
How do you think your son/daughter feels about coming to the clinic?	8 (6,10)
(0 Does not like coming; 10 Likes coming)	
How nervous do you feel when you see the team?	9 (7,10)
(0 Very nervous; 10 I do not feel nervous)	
How involved do you feel you have been in decisions made about your son/daughter’s treatment?	10 (9,10)
(0 Not at all involved; 10 Very involved)	

## Discussion

The findings from our comparative study indicate, for this restricted set of measures, that centralization of cleft services has improved parental perceptions of some child outcomes and made little if any difference to other measures. Fewer parents in the CCUK study report that their child’s self-confidence has been adversely affected by the cleft compared to those who took part in the CSAG study. There appears to have been little change in parental levels of satisfaction with 1) the appearance of the facial features in the child after surgery and 2) the provision of care within the cleft service. Parents who took part in the most recent survey report that they are satisfied with the amount of information they are given regarding their child’s treatment and also with the amount of support they receive. The centralization of services seems to have had little if any effect on the burden of attending cleft appointments – travel times are no different to those reported in 1998, and there is a similar level of problems associated with attending clinic appointments.

The reduced prevalence of perceived poor self-confidence in the children may be a function of the beneficial effects of several different factors resulting from centralization including multidisciplinary team working, improved continuity of care and increased provision of support for the family by specialist nurses and psychologists. Although the change is in a desirable direction, it is still a concern that around 1:12 parents believe that their 5-year-olds are so worried by their cleft that their self-confidence is adversely affected as a result. Our findings support those reported elsewhere in the literature for children with CLP, both from the UK and abroad. School-aged children aged six and over have been shown to have higher levels of depressive symptoms 10,14,15.There is also evidence that they are more likely to have difficulty with social relationships and to receive more negative responses than those without a visible difference 14, to be teased 10 and to have lower scores for social acceptance 16. Some authors have suggested that these adverse outcomes are associated with less optimal parenting environments 14,15,17 and that supportive clinical intervention is important, particularly around the time of school transitions 14.

The negligible changes in overall satisfaction with cleft services may be due to ceiling effects – the very high satisfaction levels in 1998 allow little room for improvement in these measures. However, items asked in the CCUK report allow us to investigate reports of parental satisfaction a little more thoroughly and it seems that some needs are being met: parents report that they are very satisfied with the support they receive from cleft team members and also with the availability and clarity of information provided regarding their child’s treatment. While such high levels of satisfaction with service are desirable, it is important to consider the pitfalls of trying to assess satisfaction with services. To begin with, the only assumption that can be made from an assessment of ‘satisfaction with service’ is that the service is adequate or acceptable: unless rated explicitly, there is no guarantee that a high level of ‘satisfaction’ means that something is judged to be ‘superior’. Also, general questions about satisfaction with service are likely to assess perceptions of service against the individual’s expectations of service rather than providing an assessment of the absolute quality of service and care provided. As a result, measures of satisfaction of service may represent a subjective rather than an objective evaluation 18 so that what satisfies one individual may not satisfy another. Indirect measures of satisfaction can be used as in the CSAG and CLAPA studies reported above: for example, how satisfied are individuals with treatment or the provision of information or support. However, in addition to the same subjectivity as described above, such indirect measures of satisfaction also pre-empt what satisfaction comprises. Discrete choice experiments provide an alternative way to quantitatively assess the provision of healthcare services 19 and facilitate a patient-centred evaluation 20. More consistent use of such objective measures may provide a more reliable assessment of the quality of service provision.

The lack of an obvious change in travel time to and problems associated with attending clinic appointments may suggest that the hub and spoke model adopted during centralization is effective: with a reduction in the number of cleft centres from 57 to 11 centres or managed clinical networks one might have predicted an increase in both travel time and problems associated with attending clinics, but this has not happened. However, it is important to note that around one-third of families still find clinic appointments less than straightforward, and efforts should be made to investigate why this is so.

There are several strengths to this study: it comprises the most recent and largest national population-based survey of children who receive their cleft treatment from cleft centres across the UK. Our findings relate to a representative sample of children from across the UK who were born with a cleft and have been on the treatment pathway after the centralization of cleft services. We have a high response rate for psychosocial, well-being and satisfaction data collected at the clinic allowing us to represent almost every family who took part in this study.

The main limitation of the study relates to data collection. Our intention was to replicate the 1998 CSAG study in order to compare child outcomes before and after centralization, but data in the CSAG and CCUK studies were not always directly comparable. The Psychology Special Interest Group (SIG) were keen to ensure that the data in the CCUK study reflected current practice and so items and response sets were modified accordingly. This highlights a tension around the potentially differing aims of data collection depending on whether the intention is to best describe current practice or to provide a direct comparison with previous research. In trying to find a way to compare data across the two studies, we acknowledge that there is a potential for our estimates of parental perceptions to be inaccurate. We have erred on the side of caution in trying to translate numeric scales into categories approximately equivalent to those used in the CSAG study and so, if anything, we are likely to have underestimated rather than over-estimated our findings. There is also an issue related to the smaller sample size for data from the health and lifestyle questionnaire that parents were given to complete at home. This questionnaire was kept separate from the RCS items in part to reduce the burden on parents during the clinic appointment. However, there was also a clinical perception that some items (on maternal well-being, parenting and parent–child relationships, to be reported elsewhere) were sensitive and therefore ought to be segregated from the standard clinical assessment. While it is true that participation in all parts of the study was completely voluntary, parents may have perceived that this questionnaire was ‘less important’ because it was not completed as part of the clinic protocol and they may therefore have made a conscious decision not to complete it. On the other hand, despite the best of parental intentions, completion of this questionnaire may simply have been over-ridden by other, more pressing matters once they returned home.

The parents who took part in the CCUK study report that their children are doing well and that they are very satisfied with the care provided by cleft services in the UK. Analysis of data from the Health and Lifestyle questionnaire will facilitate a comprehensive investigation of family relationships to improve understanding of factors associated with well-being in families where a child is born with a cleft. Ultimately, it will be possible to analyse this psychosocial data in conjunction with objective measures of clinical outcomes and service provision as reported elsewhere in this supplement and so describe associations between mental and physical well-being in these families. We may also be able to undertake longitudinal follow-up studies with this sample: this would allow us to investigate prospective associations between clinical and psychosocial outcomes at 5 years with psychosocial outcomes in late childhood and adolescence when there is an increased risk of adverse outcomes 15,21.

With respect to policy, one of the recommendations from the 1998 CSAG study was that all teams providing cleft care services should have a clinical psychologist. A survey of cleft teams and their multidisciplinary members 9 indicates that, although desirable, this recommendation is not yet reality in all centres. In our study, one in twelve parents report that their 5-year-old child has reduced self-confidence and more than a third suggested that either they or their child might want psychological support at some time in the future. There is also evidence in the literature that less optimal parenting environments might be associated with more adverse psychosocial outcomes for children away from the family context 14 reinforcing the case to provide psychological support for both parents and children in all cleft teams around the country.

## Conclusions

The current study has shown that, overall, parents are very satisfied with the provision of cleft services. One in twelve parents believe their 5-year-old child to have lower levels of self-confidence than one might hope: a smaller proportion than was reported in 1998. There appears to have been no adverse impact of centralization on families’ ability to attend clinic appointments. However, it should also be noted that there has been no reduction in the proportion of families who report difficulties in attending appointments for their child’s treatment.

## Clinical relevance

The care of children born with oro-facial clefting is complex and extends into adulthood. In 1998, findings from a national survey of 5-year-old children born with unilateral cleft lip and palate (UCLP) showed that cleft was associated with poor self-confidence at this age. As a consequence of this and other adverse outcomes, cleft services were centralized. The current study has re-assessed parental perceptions of psychosocial outcomes in 5-year-old children born with UCLP between April 2005 and March 2007 and also parental satisfaction with care. We report parental perceptions of treatment outcomes and care within a centralized service.

## References

[b1] Clinical Standards Advisory Group (1998). Cleft Lip and/or Palate, Report of a CSAG Committee.

[b2] Gorlin RJ, Cervenka J, Pruzansky S (1971). Facial clefting and its syndromes. Birth Defects Orig Artic Ser.

[b3] Jordan RE, Kraus BS, Neptune CM (1966). Dental abnormalities associate with cleft lip and/or palate. Cleft Palate J.

[b4] Williams AC, Bearn D, Mildinhall S, Murphy T, Sell D, Shaw WC (2001). Cleft lip and palate care in the United Kingdom – the Clinical Standards Advisory Group (CSAG) Study. Part 2: dentofacial outcomes and patient satisfaction. Cleft Palate Craniofac J.

[b5] Sell D, Harding A, Grunwell P (1994). A screening assessment of cleft-palate speech (Great Ormond Street speech assessment). Eur J Disord Commun.

[b6] Sell D, Grunwell P, Mildinhall S, Murphy T, Cornish TAO, Bearn D (2001). Cleft lip and palate care in the United Kingdom – The Clinical Standards Advisory Group (CSAG) study. Part 3: Speech outcomes. Cleft Palate Craniofac J.

[b7] Sheahan P, Blayney AW (2003). Cleft palate and otitis media with effusion: a review. Rev Laryngol Otol Rhinol.

[b8] Turner SR, Thomas PWN, Dowell T, Rumsey N, Sandy JR (1997). Psychological outcomes among cleft patients and their families. Br J Plast Surg.

[b9] Scott JK, Leary SD, Ness AR, Sandy JR, Persson M, Kilpatrick N (2014). Centralisation of services for children born with orofacial clefts in the United Kingdom: a cross-sectional survey. Cleft Palate Craniofac J.

[b10] Hunt O, Burden D, Hepper P, Johnston C (2005). The psychosocial effects of cleft lip and palate: a systematic review. Eur J Orthod.

[b11] Turner SR, Rumsey N, Sandy JR (1998). Psychological aspects of cleft lip and palate. Eur J Orthod.

[b12] Shaw WC, Meek SC, Jones DC (1980). Nicknames, teasing, harassment and the salience of dental features among school children. Br J Orthod.

[b13] Kramer FJ, Gruber R, Fialka F, Sinikovic B, Hahn W, Schliephake H (2009). Quality of life in school-age children with Orofacial clefts and their families. J Craniofac Surg.

[b14] Murray L, Arteche A, Bingley C, Hentges F, Bishop DVM, Dalton L (2010). The effect of cleft lip on socio-emotional functioning in school-aged children. J Child Psychol Psychiatry.

[b15] Fadeyibi IO, Coker OA, Zacchariah MP, Fasawe A, Ademiluyi SA (2012). Psychosocial effects of cleft lip and palate on Nigerians: the Ikeja-Lagos experience. J Plast Surg Hand Surg.

[b16] Broder H, Strauss RP (1989). Self-concept of early primary-school age children with visible or invisible defects. Cleft Palate J.

[b17] Despars J, Peter C, Borghini A, Pierrehumbert B, Habersaat S, Mueller-Nix C (2011). Impact of a cleft lip and/or palate on maternal stress and attachment representations. Cleft Palate Craniofac J.

[b18] Crow R, Gage H, Hampson S, Hart J, Kimber A, Storey L (2002). The measurement of satisfaction with healthcare: implications for practice from a systematic review of the literature. Health Technol Assess.

[b19] Ryan M (2004). Discrete choice experiments in health care – NICE should consider using them for patient centred evaluations of technologies. Br Med J.

[b20] Ke KM, Mackichan F, Sandy JR, Ness AR, Hollingworth W (2013). Parents’ perspectives on centralized cleft services for children: the development of a DCE questionnaire. Oral Dis.

[b21] Bos A, Prahl C (2011). Oral health-related quality of life in Dutch children with cleft lip and/or palate. Angle Orthod.

